# Why Do Some Consumers Still Prefer In-Store Shopping? An Exploration of Online Shopping Cart Abandonment Behavior

**DOI:** 10.3389/fpsyg.2021.829696

**Published:** 2022-01-20

**Authors:** Siqi Wang, Ye Ye, Binyao Ning, Jun-Hwa Cheah, Xin-Jean Lim

**Affiliations:** ^1^School of Business and Economics, Universiti Putra Malaysia, Serdang, Malaysia; ^2^Business School, Shaoguan University, Shaoguan, China; ^3^College of Economics and Management, South China Agricultural University, Guangzhou, China; ^4^Centre of Value Creation and Human Well-being, Faculty of Economics and Management, Universiti Kebangsaan Malaysia, Bangi, Malaysia

**Keywords:** wait for lower price, hesitation at checkout, perceived transaction inconvenience, online shopping cart abandonment, decision to buy from a land-based retailer, stimulus-organism-response model

## Abstract

Shopping cart abandonment remains a challenge for many e-retailers despite the continued growth of the e-commerce industry worldwide. However, the issue of online shopping cart abandonment (OSCA) has not been explored extensively in the literature. Grounded by the stimulus-organism-response (S-O-R) model, this study explores a sequential mediation model comprising consumers' wait for lower prices as an antecedent, hesitation at checkout and OSCA as mediators, perceived transaction inconvenience as a moderator, and decision to buy from a land-based retailer (DBLR) as an outcome. An online questionnaire was designed and distributed to 883 online consumers in Mainland China. Partial least squares-structural equation modeling (PLS-SEM) was employed to analyze the survey data. The results show that waiting for lower prices positively influences hesitation at checkout, and subsequently, impacts both OSCA and DBLR. Hesitation at checkout and OSCA play sequential mediating roles in the framework path. In addition, perceived transaction inconvenience strengthens the relationship between waiting for lower prices and hesitation at checkout. Overall, this study contributes to theory and serves as a guideline for e-retailers in reducing the OSCA rate.

## Introduction

By shifting many aspects of consumers' daily lives to online platforms, the COVID-19 pandemic has been an accelerator of e-commerce growth, especially that of online shopping. Research shows that about 79% of consumers preferred to order groceries online in 2020, a 19% increase from 2019 (Inmar, 2020). Although the pandemic has driven the expansion of online shopping, the rate of online shopping cart abandonment (OSCA) is estimated to be as high as 95% (Elkind, 2020), costing $4.6 trillion in lost sales (Paterson, 2020). Thus, the growth of e-commerce does not mean the demise of brick-and-mortar retail. Data shows that 84% of sales occur in physical stores and 46% of consumers still prefer to shop and interact physically with sellers (Marian, 2021). This phenomenon can be explained by the need for live experiences (e.g., viewing, touching, interacting with physical products) (33%) and immediacy (e.g., getting the item instantly) (13%), which are forms of community and connection that online experiences are always lacking (Raydiant, 2021). Another study found that 65% of consumers claim to shop in-store to avoid shipping costs (Chad, 2021). In addition, the physical stores in China offer contactless payment (Daxue, 2021) and continue to meet consumer expectations for product quality, delivery, and brand values as compared purchasing *via* online. Thus, the recovery and strategy of traditional retail make it difficult for e-commerce to compete, as physical stores in China today provides consumer with an authentic and enjoyable customer experience.

With the resurgence of brick-and-mortar retailing, scholars have developed different understandings of online consumers' “non-buyer behavior.” For example, according to Huang et al. (2018), OSCA is the final behavioral outcome that describes leaving an item in one's online shopping cart without completing the purchase. However, contrary findings are evident in the two major themes in OSCA research: purchase risk (i.e., financial risk and privacy risk) (Kukar-Kinney and Close, 2010; Xu and Huang, 2015; Kapoor and Vij, 2021) and technology-related inhibitors (i.e., website design and navigation structure) (Garaus, 2018; Kapoor and Vij, 2021). Also, recent research by Zhao et al. (2021) explored the impact of pop-up warning messages on consumers' OSCA behaviors. These studies have come to a limited understanding that consumers' OSCA behavior is not the final outcome in decision-making; rather, there could be alternative behavioral decisions following OSCA, such as consumers' decision to buy from a land-based retailer (DBLR). This phenomenon may occur due to consumers' psychological characteristics or conflicts in the decision-making process (Mishra et al., 2021). Moreover, buying from physical stores helps consumers avoid shipping and handling costs, thereby achieving a lower total cost of ownership (Kukar-Kinney and Close, 2010). Therefore, it is timely and relevant for e-retailers to understand the factors that lead to OSCA (Huang et al., 2018; Jiang et al., 2021; Mishra et al., 2021) as well as its potential effect on DBLR behavior. The main purposes of this study are: (i) to identify the drivers of OSCA and how they influence consumers' decision to purchase from physical stores; and (ii) to explore the boundary conditions that influence the relationship between consumers' wait for lower prices and hesitation at checkout.

Although previous studies have shown that the price factor is a key inhibitor of purchase behavior (Kukar-Kinney and Close, 2010; Song, 2019), this notion has not been fully explored in the contexts of both OSCA and DBLR. Rajagopal (2019) defined price as an influential factor in consumers' preference, perception of value for money, purchase intention, consumption experience, and behavior. To some extent, price fluctuations can delay consumers' purchase decisions, which results in their hesitation to checkout (Kukar-Kinney and Close, 2010). This effect is said to be increasingly critical, especially during the COVID-19 pandemic when consumers have been expected to reduce unnecessary spending to better cope with future uncertainty and risk (Jin et al., 2021). Thus, it seems a pertinent direction to examine consumers' perceived prices (i.e., their wait for lower prices) as the antecedent that influences hesitation at checkout and OSCA, thus resulting in DBLR.

Hesitation at checkout, in turn, is perceived as consumers' espousal of additional processing time to delay purchases before making a final purchase decision online (Cho et al., 2006). A consumer's hesitation during the purchasing process can lead to an unpleasant motivational state that postpones decision-making (Huang et al., 2018). The various risks associated with online shopping (e.g., financial risk, product risk, and time loss) (Demirgüneş, 2018) form a sense of hesitation toward online shopping, which leads to OSCA (Huang et al., 2018). While the impact of checkout hesitancy on OSCA has been well-documented (Cho et al., 2006; Huang et al., 2018), earlier work has ignored the former's potential impact on purchasing from the offline channel (i.e., DBLR), especially due to the COVID-19 pandemic. Therefore, this study extends the work of Huang et al. (2018) by exploring the sequential mediating effects of both hesitation at checkout and OSCA on the linkage between waiting for lower prices and DBLR.

Motivated by several reasons, this study also examines the moderating role of perceived transaction inconvenience. First, convenience is one of the most important predictors of a consumer's choice to shop online (Childers et al., 2001; Sembada and Koay, 2019). For example, a study by Tandon et al. (2016) highlighted that consumers' willingness to purchase online depends on the convenience of the website. Another study by Raman (2019) showed that the convenience of transactions is an important variable in forming consumers' positive attitudes and predicting their willingness to purchase online. Second, convenience makes an important contribution to the value of consumers' desired outcome (Sembada and Koay, 2019). Prior research has shown that when transactions become complex (e.g., lengthy registration forms, technical glitches, complex discount rules), consumers are more inclined to abandon their shopping carts due to the challenging buying process (Rajamma et al., 2009). In consideration of this evidence, another aim of this study is to explore the potential of perceived transaction inconvenience as the conditioning factor affecting hesitation to checkout.

The remainder of the study is organized as follows. First, the theoretical background is discussed and the relevant literature on price and the hesitation mindset is reviewed. Next, the research framework and hypotheses are developed, after which the process of data collection and the final sample size are described. Fourth, the results of the data analysis are presented. Then, the findings are discussed, based on which theoretical and practical implications are presented. Finally, the study's limitations are outlined and suggestions for future research are offered.

## Theoretical Background and Hypotheses

### Stimulus-Organism-Response Model

This study adopts the S-O-R framework as its overarching theory. As a widely used theory in the consumer behavior literature (Zheng et al., 2019; Akram et al., 2020; Arif et al., 2020; Paz and Delgado, 2020; Lim et al., 2021; Wang et al., 2022), this model emphasizes how environmental stimuli lead to cognitive and affective responses (i.e., perception, experience, and evaluation), which in turn trigger certain psychological responses (i.e., attitudinal and behavioral responses) (Mehrabian and Russell, 1974).

In the domain of online selling, stimuli include websites, advertising, products, packaging, value, convenience, and entertainment (Hsu et al., 2012; Wong et al., 2012), all of which are known to influence consumers' emotions during the purchase journey (Jacoby, 2002). A great number of empirical studies anchored in the S-O-R model have confirmed that price is a significant stimulus that influences consumers' online and offline purchase behavior. For example, the study by Chen and Yao (2018) showed that consumers give positive feedback and tend to make impulse purchases when the price of a product is reduced. Furthermore, Cheah et al. (2020b) highlighted that price image has a huge impact on consumer perceptions, including perceived value, trust in retailers, and attitudes. More recently, research by Lee et al. (2021) identified that price attributes have a positive effect on perceived enjoyment as a predictor of consumer purchase behavior. Furthermore, the buyer behavior theory (Howard and Sheth, 1969) of price factor was extended to the online environment as an inhibitor, showing the impact of price on consumer shopping cart abandonment (Kukar-Kinney and Close, 2010). Thus, the empirical evidence strongly suggests that price is an important stimulus that affects consumer emotion and behavior.

Next, the “organism” is the undertaking of emotional, psychological, and cognitive internal processes between the stimulus and the response (Han and Kim, 2020). In the domain of marketing, factors such as satisfaction, emotion, and self-confidence are considered as “organisms” that impact consumers' purchase intentions. For example, as recommended by Yuan et al. (2020), satisfaction can be used to measure the impact of consumer loyalty on mobile payment providers. Chang et al. (2011) study demonstrated that consumers with more positive emotional responses to retail environments and retailer are more likely to make impulse purchases. In addition, Cham et al. (2020) identified the positive impact of self-confidence on purchase decisions. Notably, Huang et al. (2018) measured the strong influence of checkout hesitation on OSCA behavior. That is, hesitation can be viewed as a decision-making style that appears in the process of exploring, alerting, and analyzing (Demirgüneş, 2018). Its impact is more significant before the consumer makes a final purchase decision (Peng and Chen, 2019). To further validate these early assumptions, this study echoes hesitation at checkout as the “organism” that impacts consumers' online purchase decisions, including OSCA and DBLR.

**Figure 1:**
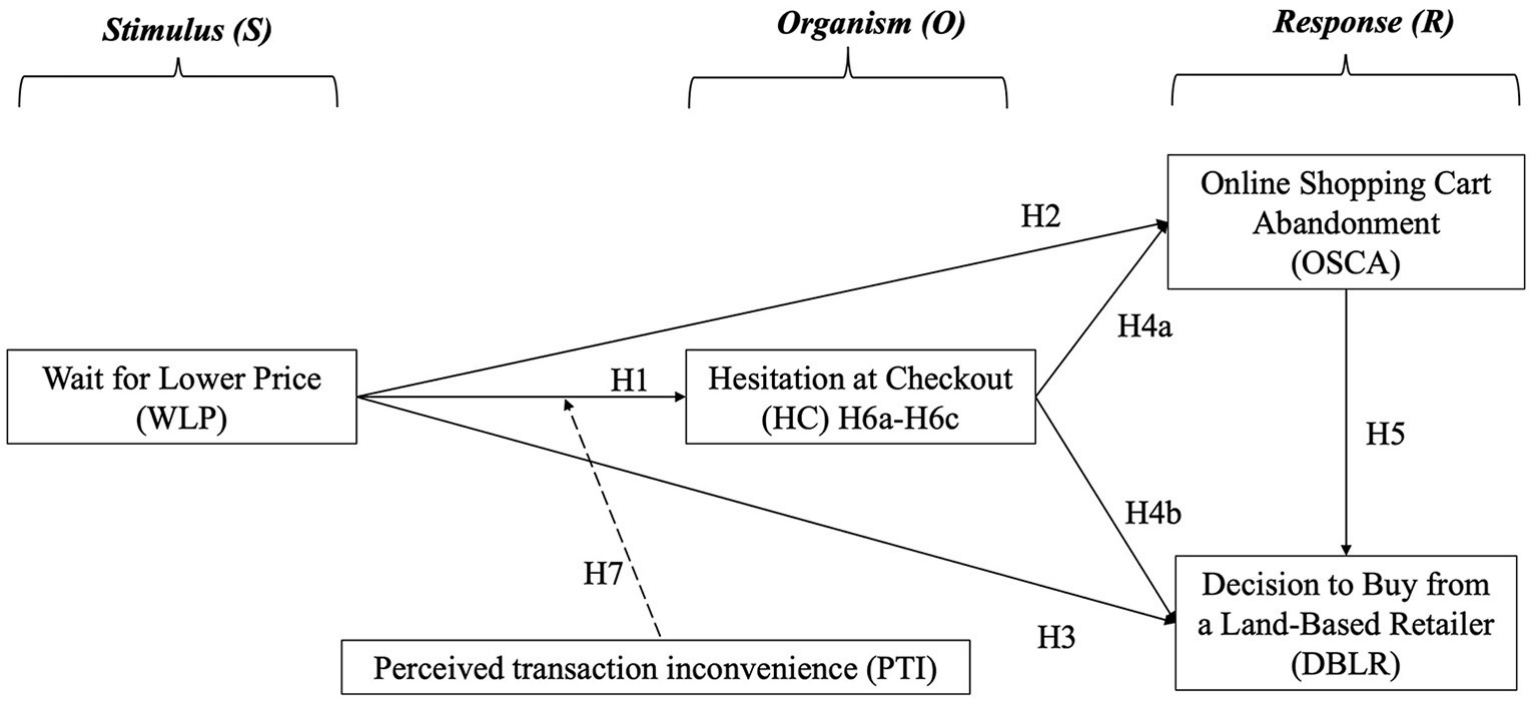
Research model.

Taken together, our study enriches the existing literature by considering consumers' wait for lower prices as a stimulus that triggers their checkout hesitation “organism,” while OSCA and DBLR are the target “responses” we seek to explore. To extend the applicability of the S-O-R model, we also incorporate perceived transaction inconvenience as a moderator that strengthens the magnitude of hesitation to checkout. The research model is shown in Figure 1. The hypothesized relationships between the proposed constructs are clarified in the following sections.

### Impacts of Consumers' Wait for Lower Price

In online shopping, consumers are always eager to find the best deals, as price serves as an attractive stimulus in this environment (Kukar-Kinney and Close, 2010; Han et al., 2020). According to Kukar-Kinney and Close (2010), the wait for lower price concept is defined as “consumers' decision to wait until a lower price can be found on at least some item(s) in the cart, whether it be at the same or a different store, through the same or a different channel” (p. 244).

It has been some time since scholars reached the consensus that price awareness is a critical factor influencing consumer attitudes and behaviors (Zhang et al., 2020). Coppola and Sousa (2008) stated that price consciousness is the strongest predictor of overall shopping hesitancy. This claim is supported by Azimi, Milne and Miller (2020) finding that price consciousness increases the likelihood of procrastination and exit. This evidence seems to suggest that when consumers plan to wait for a sale or the lowest price, they are most likely to exhibit hesitation in buying (H1). Similarly, Nelson et al. (2007) demonstrated that the dynamics of price change prompt consumers to always check different stores or shop at different times. In addition, Kukar-Kinney and Close (2010) confirmed that waiting for lower prices is a key factor that increases the OSCA rate. Thus, this study hypothesizes that when consumers intend to wait for lower prices, the more likely they are to engage in OSCA (H2).

The outbreak of the COVID-19 pandemic has further led to a low level of consumer spending. Ellison et al. (2021) argued that many households are still facing financial deficiencies that may push them to switch to low-cost retailers. The studies by both Dahana et al. (2018) and Tu and Zhou (2012) confirm that consumers choose different purchase channels due to concerns about lower prices. In the case of an unexpected public health event, waiting for lower prices significantly increased the decision to buy from land-based retailers (H3). Therefore, supported by the S-O-R model, this study considers waiting for lower prices in online to be a significant stimulus that triggers hesitation at checkout as well as two different purchase decisions (i.e., OSCA and DBLR). The hypotheses are proposed as follows:

H1: The wait for lower prices positively affects hesitation at checkout.
H2: The wait for lower prices positively affects OSCA.
H3: The wait for lower prices positively affects DBLR.

### Drivers of OSCA and DBLR

Cho et al. (2006) defined hesitation at checkout as “postponing or deferring product purchases by having additional processing time before making final product-purchases on the Internet” (p. 261). Research by Jessup et al. (2009) clarified that the multiple choices or uncertainties present in online shopping can lead consumers to leave empty-handed. In particular, consumers terminate transactions most likely because they are hesitant (Huang et al., 2018). Likewise, Tian et al. (2019) concluded that consumers who have a “whether to buy” mindset have a greater willingness to abandon their purchase. These outcomes suggest that consumers who experience hesitation at checkout are more likely to engage in OSCA behavior (H4a). At the same time, hesitant consumers are more likely to purchase offline so as to mitigate the uncertainty risks associated with online purchases (e.g., product return risk, payment security risk, time loss) (Klepek and Bauerová, 2020). Therefore, we hypothesize that consumers with hesitation at checkout are more likely to turn toward DBLR (H4b).

Empirical evidence has confirmed that consumers choose different purchase channels to maximize their shopping benefits (Verhoef et al., 2015; Gensler et al., 2017). For instance, information overload in the searching stage is the main inhibitor of online shopping, driving consumers to switch toward offline purchases (Walsh and Mitchell, 2010) where they can see and touch what they want (Flavián et al., 2020). That is, consumers with a need for human interaction will move from online to offline shopping platforms as a way to mitigate the perceived uncertainty of online transactions (Aw, 2020). Additionally, brick-and-mortar stores save on delivery time by offering the immediate possession of items, which is an advantage often sought by consumers (Wollenburg et al., 2018). With this in mind, consumers are more likely to abandon their online shopping carts and show interest in land-based retailers to achieve their purchase needs (H5). Therefore, this study proposes the following hypotheses:

H4a: Hesitation at checkout positively affects OSCA.
H4b: Hesitation at checkout positively affects DBLR.
H5: OSCA positively affects DBLR.

### Mediation Effects

The empirical evidence suggests that price influences consumers' purchase decisions (Büyükdag et al., 2020). Meanwhile, Cho et al. (2006) showed that checkout hesitation, due to key reasons like uncertainty, perceived risk, or additional information, affects consumer behavior. As a result, purchases are often delayed in the pursuit of lower prices and the search for merchants that offer a discount (Bauer et al., 2006). When checkout hesitation occurs, consumers are more likely to end their shopping process and leave the item(s) in the shopping cart (Huang et al., 2018). We therefore hypothesize that when consumers wait for lower prices, they may hesitate at checkout, leading to an increased likelihood of their DBLR (H6a). Based on this reasoning, we propose the following hypothesis:

H6a: Hesitation at checkout mediates the relationship between the wait for lower prices and DBLR.

Consumers perceive hesitation about their freedom of purchase choice when unexpected circumstances hinder their attitudinal or behavioral responses (Wong and Yeh, 2009). Hesitation is the most common problem in online purchasing because of the increased likelihood of consumers' exposure to risk (e.g., financial risk, product risk, and time loss) (Demirgüneş, 2018). In this context, it has been mentioned that consumers' feelings of hesitation that can lead to unpleasant motivational outcomes (Schrift et al., 2011). For example, consumers who hesitate at the checkout point are more likely to abandon their mobile shopping cart (Huang et al., 2018). We argue that shopping at a physical store can mitigate the risk of uncertainty that is inherent in online shopping. Therefore, consumers with checkout hesitancy are more likely to prefer on land-based retail shopping after abandoning their online shopping cart (H6b). We thus hypothesize that:

H6b: OSCA mediates the relationship between hesitation at checkout and DBLR.

From H6a and H6b, our study also suggests that hesitation at checkout and OSCA may play a sequential mediating role between the wait for lower prices and DBLR. Based on the S-O-R model, when consumers choose to wait for lower prices, they are more likely to have checkout hesitation, which leads to both OSCA and DBLR behavior. Therefore, in this study, we take a step further by exploring hesitation at checkout and OSCA as sequential mechanisms that potentially connect the path between the wait for lower prices and DBLR (H6c). The hypothesis is proposed as:

H6c: Hesitation at checkout and OSCA sequentially mediate the path between the wait for lower prices and DBLR.

### Moderating Effect of Perceived Transaction Inconvenience

Convenience refers to “the extent to which a customer feels that the website is simple, intuitive, and user friendly” (Srinivasan et al., 2002, p. 44). Earlier e-commerce research has highlighted that when a transaction is accompanied by a bothersome process, it will lead to delay, switching, and even abandonment (Corsten and Gruen, 2003; Gunasti and Ross, 2009). Accordingly, a convenient website can accelerate online consumers' willingness to shop (Raman, 2019), whereas if consumers are hindered and frustrated during the transaction, they are less likely to return (Cameron, 1999).

Rajamma, Paswan and Hossain (2009) study extended the concept of convenience by proposing perceived transaction inconvenience (i.e., lengthy registration forms, technical glitches, etc.) as an important inhibiting factor that affects OSCA by complicating transactions and increasing consumers' frustration. Xu and Huang (2015) also highlighted that perceived transaction inconvenience (e.g., slow loading of web pages, complex transaction process, etc.) affects consumers' access to the shopping cart usage stage. Consumers who feel inconvenienced when accessing useful and direct information from a website would have a lower level of trust and subsequent willingness to visit online stores (Chen and Barnes, 2007). Indeed, it has been suggested that perceived transaction inconvenience decreases consumer trust in social media stores and exerts a significant impact on shopping intentions (Sembada and Koay, 2019). Based on this evidence, perceived transaction inconvenience would moderate the relationship between the wait for lower price and hesitation at checkout (H7). Therefore, we propose that:

H7: When perceived transaction inconvenience is high, the relationship between the wait for lower price and hesitation at checkout is stronger.

## Methodology

### Data Collection and Sampling

With over 872 million people engaging in online shopping, China has become the second-largest e-tailing market in the world after the United States (Statista, 2021a). However, many retailers still prefer to expand their brick-and-mortar stores, on the basis that the live experience attracts more consumers as a competitive advantage (Marian, 2021). Furthermore, a recent report by Statista (2021b) documented that as of December 2020, despite ~79.1% of Chinese consumers purchasing on various shopping platforms (e.g., Taobao, Tmall, and JD.COM), the OSCA rate was about 76.3% (Creditdonkey, 2019). One of the product categories with the highest OSCA rate is women's clothing (FinancesOnline, 2021). Evidently, empirical research on respondents with extensive Internet coverage and experience in online shopping is highly appropriate. Therefore, Mainland China was the study site for data collection in this study.

To collect the data, an online survey form was created through wenjuanxing (https://www.wjx.cn), one of the largest online survey platforms in China. The questionnaire was sampled using purposive sampling as this method is viewed effective in obtaining valid responses which could provide information relevant to the study (Saunders et al., 2012). In this study, respondents who are Chinese from Mainland China (Southern and Northern China) and have an online shopping experience are invited to answer the survey between January and March 2021. Since this study was targeted at online consumers in Mainland China, the questionnaire was designed in English and back-translated into Chinese to ensure all the items expressed the same meaning (Brislin, 1970). Before formal data collection, the study instrument was pre-tested by a panel of five individuals, comprising practitioners, academics, and target respondents, to comment on the representativeness and applicability of the questionnaire. Afterwards, a pilot test was conducted with 30 respondents who fit the research context. Based on their feedback, the final measurement items underwent minor changes to their wording and layout (see Appendix 1).

After excluding one response with obvious regularity, a total of 883 valid responses were retained. According to Hair et al. (2019), a sample size of 883 is considered adequate; this number also exceeded the minimum sample size required for *post-hoc* analysis^1^. The demographic profile of the study participants was analyzed using frequency tests. The results showed that most of the respondents were aged between 21 and 30 years old (37.30%), female (58.60%), bachelor's degree holders (55.30%), earned a monthly income of less than RMB 5,000 (75.80%), and from the northern region (52.92%). Additionally, a majority of the respondents indicated that they have 1–3 years of experience with online shopping (41.00%) (see Table 1).

**Table 1:** Respondent profile (*N* = 883).

**Demographic profile**	**Category**	**Frequency**	**Percent (%)**
Gender	Male	366	41.40
	Female	517	58.60
Age	20 years old and below	327	37.00
	21–30 years old	329	37.30
	31–40 years old	100	11.30
	41–50 years old	78	8.80
	51 years old and above	49	5.50
Monthly income	Below 5,000	669	75.80
	5,001–7,000	90	10.20
	7,001–9,000	49	5.50
	9,001–11,000	38	4.30
	11,001–13,000	14	1.60
	13,001–15,000	7	0.80
	15,001–17,000	3	0.30
	17,001 and above	13	1.50
Education	Less than High School	34	3.90
	High School Diploma	51	5.80
	College Degree	196	22.20
	Bachelor's degree	488	55.30
	Master's Degree	99	11.20
	Doctorate (PhD or equivalent)	15	1.70
Residence	Southern China	416	47.10
	Northern China	467	52.90
Online Shopping Years	Less than 1 year	47	5.30
	1–3 years	362	41.00
	4–6 years	322	36.50
	7–9 years	88	10.00
	10 years or more	64	7.20

### Measures

All items measured in this study were based on validated and reliable multi-item scales drawn from previous research, with some modifications to accommodate the current research setting. Wait for lower prices and OSCA were measured using the scales developed by Kukar-Kinney and Close (2010); hesitation at checkout was adapted from Cho, Kang and Cheon (2006) and Wong and Yeh (2009) scales; DBLR was measured using Rapp, Baker, Bachrach, Ogilvie and Beitelspacher (2015) scale; and finally, the scale for perceived transaction inconvenience was adapted from Rajamma et al. (2009). All these measurements have demonstrated great validity and reliability results, which is the reason for their inclusion in this study.

## Data Analysis

Two types of statistical software were used to analyze the data in this study. First, the statistical software SPSS version 28 was used to assess demographic frequencies and check for common method bias (CMB). Subsequently, partial least squares-structural equation modeling (PLS-SEM) using SmartPLS version 3.3.3 (Ringle et al., 2015; Sarstedt and Cheah, 2019) was employed to examine the hypothesized relationships. This technique has become a quasi-standard in the marketing field, especially when evaluating complex relationships between various latent variables (i.e., mediation and moderation) and simultaneously examining the relationships between variables in terms of explained variation (Hair, 2020). Additionally, PLS-SEM is appropriate when the research goal is prediction-oriented and/or when the research is exploratory by nature (Cheah et al., 2019; Chin et al., 2020; Hwang et al., 2020).

### Common Method Bias

Since the design of this study adopted a cross-sectional approach, two CMB assessments were performed: Harman's single factor (Podsakoff et al., 2003) and the full collinearity test (Kock and Lynn, 2012). First, the results of Harman's single factor test illustrated that the variance explained by the first factor was 36.036% (<40%), indicating that CMB was not a concern in this study (Babin et al., 2016). Second, the full collinearity test showed that the variance inflation factor (VIF) values were in the range of 1.247–2.038 (<3.33) (refer to Table 2), signifying CMB does not pose any severe issue in this study (Kock and Lynn, 2012).

**Table 2:** Results of the measurement model.

**Construct**	**Item**	**Loading**	**VIF**	**CA**	**rho_A**	**CR**	**AVE**
Wait for lower prices	WLP1	0.858	1.229	0.604	0.643	0.793	0.568
	WLP2	0.801					
	WLP3	0.572					
Hesitation at checkout	HC1	0.816	2.054	0.913	0.914	0.935	0.742
	HC2	0.849					
	HC3	0.887					
	HC4	0.878					
	HC5	0.876					
Online shopping cart abandonment	OSCA1	0.828	1.968	0.813	0.819	0.878	0.645
	OSCA2	0.861					
	OSCA3	0.822					
	OSCA4	0.690					
Decision to buy from a land-based retailer	DBLR1	0.831	1.573	0.859	0.876	0.903	0.699
	DBLR2	0.866					
	DBLR3	0.850					
	DBLR4	0.796					
Perceived transaction inconvenience	PTI1	0.661	1.142	0.712	0.718	0.822	0.537
	PTI2	0.720					
	PTI3	0.809					
	PTI4	0.734					

*VIF, variance inflation factor; CA, convergent validity; CR, composite reliability; AVE, average variance extract*.

### Assessment of the Measurement Model

Table 2 exhibits the reflective measurements' assessment in terms of their reliability and convergent validity. Regarding the outer loading, all items met the recommended outer loading criterion (i.e., between 0.572 and 0.887) (Hair et al., 2019). As for internal reliability, all metrics (CA, rho_A, and CR) demonstrated values above the critical value of 0.7; thus, all the measurements were internally consistent (Hair et al., 2019). In terms of the AVE, all the constructs reported values above 0.5 (Hair et al., 2019), establishing their convergent validity.

In addition, we assessed discriminant validity using the heterotrait-monotrait ratio of correlations (HTMT). Based on Table 3, all the HTMT values were lower than the conservative threshold value of 0.85 (Henseler et al., 2015). Thus, it can be concluded that discriminant validity was established between all the constructs.

**Table 3:** Discriminant validity test using HTMT criterion.

**Construct**	**1**	**2**	**3**	**4**	**5**
1. Decision to buy from a land-based retailer					
2. Hesitation at checkout	0.420				
3. Perceived transaction inconvenience	0.480	0.430			
4. Online shopping cart abandonment	0.543	0.803	0.457		
5. Wait for lower prices	0.439	0.545	0.555	0.518	

*HTMT <0.85 (Henseler et al., 2015)*.

### Assessment of the Structural Model

The assessment of the structural model started by evaluating the collinearity between the predictors. Table 4 exhibits that the VIF values of all the combination paths—which ranged from 1.144 to 2.052—were below the threshold of 3.33 (Becker et al., 2015). Hence, collinearity between the predictors was not an issue in this dataset.

**Table 4:** Structural model results.

						**BCa 95% CI**					
	**Hypothesis**	**Std. beta**	**Std. error**	***t*-value**	***p*-value**	**LB**	**UB**	**VIF**	***f^2^***	***R* ^2^**	***Q* ^2^**
Direct effect	H1: WLP -> HC	0.326	0.035	9.478	0.000	0.279	0.387	1.144	0.123	0.237	0.169
	H2: WLP -> OSCA	0.098	0.029	3.422	0.000	0.045	0.144	1.209	0.016	0.491	0.315
	H3: WLP -> DBLR	0.136	0.040	3.464	0.000	0.071	0.205	1.228	0.021	0.240	0.152
	H4a: HC -> OSCA	0.654	0.026	25.286	0.000	0.611	0.696	1.209	0.697		
	H4b: HC -> DBLR	0.090	0.052	1.657	0.049	0.009	0.170	2.052	0.005		
	H5: OSCA -> DBLR	0.350	0.050	7.062	0.000	0.271	0.425	1.966	0.083		
Mediating effect	H6a: WLP -> HC -> DBLR	0.028	0.017	1.648	0.100	−0.006	0.062				
	H6b: HC -> OSCA -> DBLR	0.230	0.034	6.790	0.000	0.167	0.295				
	H6c: WLP -> HC -> OSCA-> DBLR	0.074	0.013	5.924	0.000	0.052	0.101				
Moderating effect	H7: WLP*PTI -> HC	0.106	0.054	1.963	0.017	0.036	0.197		0.021		

*WLP, wait for lower prices; HC, hesitation at checkout; OSCA, online shopping cart abandonment; DBLR, decision to buy from a land-based retailer; PTI, perceived transaction inconvenience*.

Based on Table 4, wait for lower prices revealed a positive relationship with hesitation at checkout (β = 0.326; *p* < 0.000), supporting H1. The effects of the wait for lower prices on OSCA (β = 0.098; *p* < 0.000) and DBLR (β = 0.136; *p* < 0.000) were also found to be positive and significant; hence, both H2 and H3 were supported. Similarly, hesitation at checkout positively impacted both OSCA (β = 0.654; *p* < 0.000) and DBLR (β = 0.090; *p* < 0.05) in this study, supporting H4a and H4b. Finally, OSCA (β = 0.350; *p* < 0.000) showed a positive impact on DBLR, confirming H5.

From these findings, the R^2^ results from H1, H2, and H3 were ~23.7, 49.1, and 24.0%, respectively. To assess the significance of each path, the findings of effect size (*f*^2^) were also reported. Based on Table 4, the hypothesized paths of H1, H3, and H5 carried a small (*f*^2^ ranged from 0.021 to 0.123) but meaningful effect (Cohen, 1988). For the hypothesized path H4a, the results showed a large effect size (*f*^2^ = 0.697), while the paths of H2 and H4b exhibited a trivial effect size with values <0.02.

Finally, the predictive relevance of the model was evaluated using the blindfolding procedure (Shmueli et al., 2019). The *Q*^2^ values for all endogenous constructs in Table 4 were greater than zero, verifying the model's predictive relevance.

### Mediating Analysis

To account for the mediation roles of hesitation at checkout and OSCA, we used the method of Aguinis et al. (2017) and Nitzl et al. (2016). As can be seen in Table 4, the proposed mediation effects of OSCA (H6b) (β = 0.230; *p* < 0.000) and the sequential mediation (H6c) (β = 0.074; *p* < 0.000) exhibited a significant indirect effect with full mediation, while hesitation at checkout (H6a) failed to show significant mediation.

### Moderating Analysis

**Figure 2:**
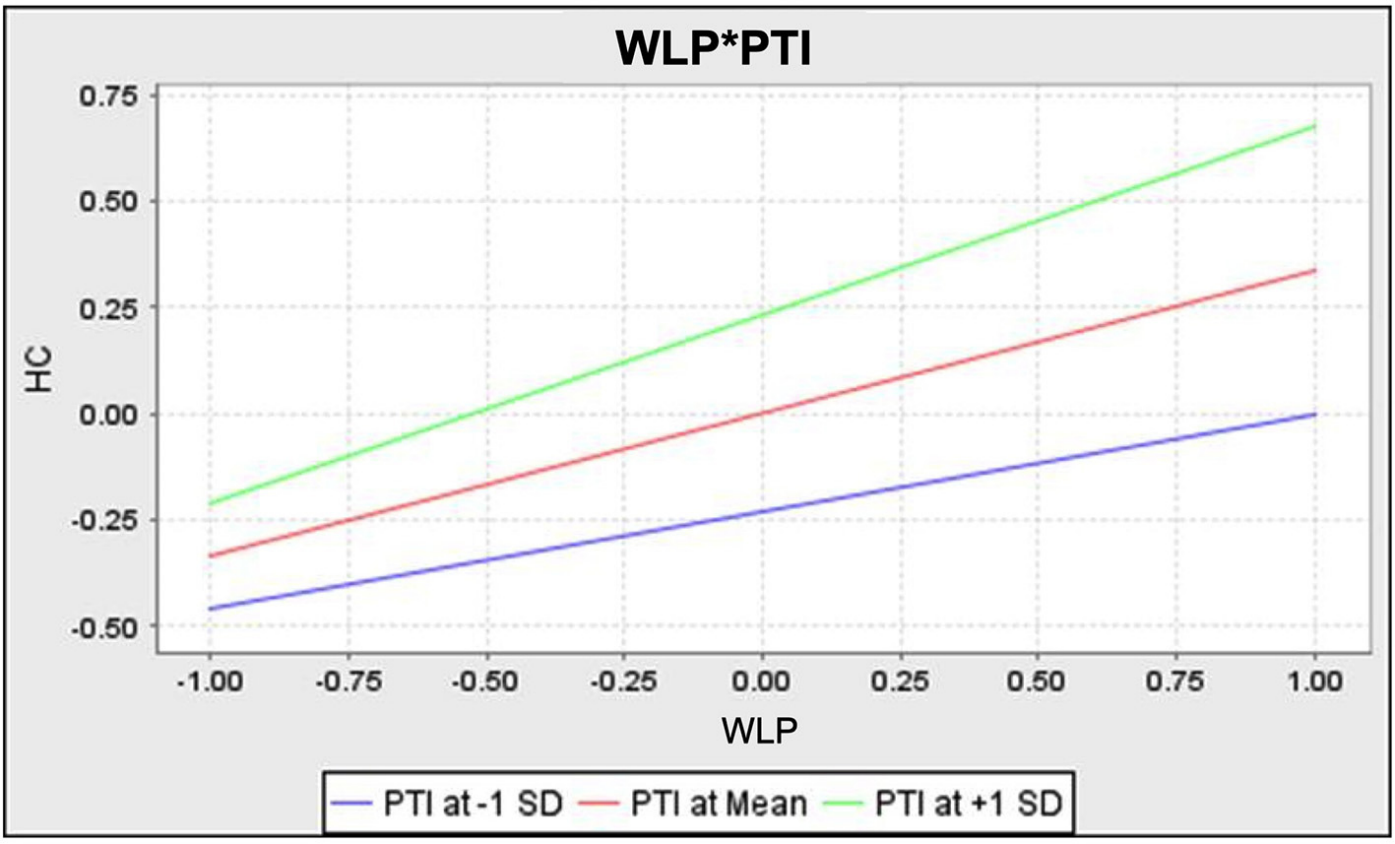
Interaction plot of WLP*PTI on hesitation at checkout.

Finally, a two-stage approach was used for the moderation analysis (Becker et al., 2018). As shown in Table 4, perceived transaction inconvenience was found to moderate the relationship between the wait for lower prices and hesitation at checkout (β = 0.101, *p* < 0.05); thus, H7 was supported. To corroborate these findings, we also assessed both the effect and the interaction plot (Dawson, 2014). The results reported that H7 had a small effect (*f* ^2^ = 0.021) (see Table 4). The interaction plot, on the other hand, indicated that the line of high perceived transaction inconvenience has a steeper gradient than low perceived transaction inconvenience (see Figure 2). This indicates that when consumers perceive high transaction inconvenience during online shopping, the positive relationship between the wait for lower prices and hesitation at checkout is stronger.

## Discussion

### Findings

This study aimed to explore the antecedents that impact OSCA and DBLR among online consumers in Mainland China. Our findings exhibit that waiting for lower prices affects consumers' internal cognitive process (i.e., hesitation at checkout), which in turn influences their purchase decision (i.e., OSCA, DBLR). Perceived transaction inconvenience is evidenced as a conditional effect that reinforces consumers' OSCA behavior. This study provides empirical support for these findings with a strong foundation anchored in our framework constructed using the S-O-R model.

First, we found that the wait for lower prices positively influences hesitation at checkout, OSCA, and DBLR (H1, H2, and H3 were supported). This finding suggests that price incentives substantially interfere with consumers' cognitive state and purchase decisions in online shopping. Confirming previous research, prices listed on online stores influence consumers' decisions, whereby those with high value consciousness are more likely to delay online purchases (Cho et al., 2006) and eventually choose to select items in physical stores (Kukar-Kinney and Close, 2010).

In addition, hesitation at checkout has the greatest impact on consumers' abandonment of their online shopping carts (H4a was supported). This finding confirms Huang, Korfiatis and Chang (2018) postulation that consumers who hesitate at checkout are more likely to abandon their shopping carts. Meanwhile, we extended the study by Huang et al. (2018) to highlight the relationship between hesitation at checkout and the decision to buy from a land-based retailer. The results of the study suggest that hesitation at checkout has a positive direct impact on DBLR (H4b was supported). Suggesting that most online consumers in Mainland China prefer to visit brick-and-mortar stores in the post-pandemic period as they have the chance to physically touch and feel items before committing to a purchase. In addition, this supports the earlier findings of Kukar-Kinney and Close (2010) that consumers choose physical stores after abandoning their online shopping carts (H5 is supported), which demonstrates that consumers who abandon their cart are very likely to re-pick similar items from physical outlets to reduce worries and risks from online purchasing.

Next, the existing literature highlights those consumers are readily abandoning their shopping carts because they are value-conscious (Mishra et al., 2021). Meanwhile, hesitant consumers are more likely to end their shopping process and leave the item in their online shopping cart (Huang et al., 2018). This study used hesitation at checkout and OSCA as sequential mediators to assess the effect between the wait for lower prices and DBLR. The results show that two indirect effect hypotheses were supported (H6b, H6c), while H6a was not. This finding supports the study of Kukar-Kinney and Close (2010) that extends the roles of hesitation at checkout and OSCA as mediating variables that explain the overall proposed S-O-R model.

Finally, the findings show that perceived transaction inconvenience is a moderator that strengthens the relationship between the wait for lower prices and hesitation at checkout (H7 was supported), due to the fact that consumers' high perceived transaction inconvenience creates expectation uncertainty (Harrison-Walker, 2002; Rajamma et al., 2009). Thus, our results extend previous arguments in the literature and consolidate our understanding of how perceived transaction inconvenience leads to OSCA behavior (Rajamma et al., 2009).

### Theoretical Implications

The results of the study provide numerous theoretical contributions. First, the present study enriches the applicability of the S-O-R model in OSCA research. Specifically, it adds to the literature on consumer purchase decisions by investigating the wait for lower prices as a stimulus. A large number of scholars have confirmed that price affects consumers' purchase intention (Coppola and Sousa, 2008; Azimi et al., 2020; Zhang et al., 2020) across different selling platforms. However, there have been no in-depth studies on how price factors affect OSCA and DBLR, especially after the outbreak of COVID-19. In this study, we found that waiting for lower prices is an important antecedent of consumers' choices pertaining to hesitation at checkout, OSCA, and DBLR. This finding echo that of Kukar-Kinney and Close (2010), highlighting the important role of price factors in consumer purchase decisions. Next, this study establishes hesitation at checkout as an “organism” that produces different responses (i.e., OSCA and DBLR) under the influence of stimuli. Extending the study of Huang et al. (2018), we enhance the value of hesitation at checkout in existing research with support from the S-O-R model.

Second, this study moves beyond direct relationships to examine hesitation at checkout and OSCA as sequential mediators that elucidate the linkage between the wait for lower prices and DBLR. The findings support our proposed hypotheses that consumers delay their purchase behaviors due to concerns about seeking lower prices, which leads to OSCA and eventually, their decision to buy the selected items from a physical store. This sequential mediation path does not only complement the conclusions drawn in the study of Kukar-Kinney and Close (2010) but also offers a comprehensive understanding for marketing researchers, particularly in the OSCA and DBLR literature.

Finally, based on the present results, perceived transaction inconvenience can be integrated as a boundary condition in addressing the OSCA issue. The moderation analysis shows that when consumers perceive higher transaction inconvenience, the positive relationship between the wait for lower prices and checkout hesitation becomes stronger. It can subsequently be concluded that the likelihood of consumers' abandoning their online shopping cart is high when transaction inconvenience arises. This outlines an important concern for future research which intends to understand the factors that influence hesitation to checkout and OSCA.

### Practical Implications

Our findings also provide meaningful implications to reduce the OSCA phenomenon. First, the significant impact of the wait for lower prices on hesitation at checkout, OSCA, and DBLR suggests that price is a key factor in consumers' decision to buy from either online or offline stores. Consumers tend to wait for items to be discounted or go to different online stores to compare prices. Ultimately, they may even choose a physical store for price-related reasons, such as to avoid shipping fees. Therefore, e-retailers should take effective measures to bridge the gap between pricing and OSCA. Hidden price promotions can be one of the strategics to address the issues, where final prices are not revealed to consumers when they first encounter a product (Li et al., 2022). Through this strategy, customers viewing the product page will focus their attention on the product's information, features, and benefits, rather than just its price (Hoang, 2020). Indeed, improving customer experience in this manner can help motivate visits and increase the likelihood of potential consumers adding the product to their shopping cart. In addition, by offering higher discounts, e-retailers can increase their prospects of meeting or exceeding consumers' expectations of price. Apart from that, e-retailers should also consider implementing a daily low-price strategy and flash sales at different periods as a way to boost online sales. More importantly, our study accounts for consumers' DBLR behavior by urging e-retailers to offer real financial compensation in their offline stores in the form of coupons, cashback, free samples, and more. This is beneficial for consumers who tend to go to brick-and-mortar stores.

Second, by observing checkout hesitation's significant impact on consumers' purchase decisions, e-retailers should develop strategies to reduce such feelings of hesitation. For example, it is important to provide more details on product descriptions and enhance search engine optimization on product pages to eliminate the feeling of hesitation. Next, e-retailers should hire experienced sellers to answer consumer inquiries and improve timely responses during the online shopping process. In addition, they can consider offering “warm” strategies (i.e., first-person narration, “I” or “you”) for consumers to provide 24/7 support services and increase interaction with consumers (e.g., using chatbots, voice recognition, and live streaming video) (Lim et al., 2021). This measure could help consumers move quickly to the payment stage, which would ultimately reduce OSCA issues. For consumers who prefer brick-and-mortar stores, retailers can implement offline smart retailing in their physical stores. For example, through digital transformation (e.g., electronic price tags) and intelligent empowerment (e.g., robot guidance), physical stores can become more intelligent and firmly “stick” consumers offline.

Finally, this study unearths new evidence on perceived transaction inconvenience, signifying an important idea in reducing the OSCA phenomenon. Often, transaction inconvenience in online shopping is mainly due to the lack of flexibility, such as the need to fill out lengthy registration information forms (Rajamma et al., 2009). E-retailers should be sympathetic to the needs of consumers and reduce these negative reactions by providing basic information storage features, online self-service options, and easier initiation of transaction processes. E-retailers can also intervene to build trust through the introduction of external links. For example, they can insert links to partner platforms (e.g., Whatsapp, WeChat) on their sales site to establish positive partnerships, such as by regularly interacting with consumers, sending item shipment progress details, or promoting the goodwill of their transactions. When consumers are aware of the controllability of an operation, their perceived transaction inconvenience will weaken. E-retailers should thus strive to capture behavioral cues from consumers to ensure that the latter are gently pushed down the sales funnel to conversion.

### Conclusion, Limitations and Future Research

Based on the S-O-R model, this study examined the factors that influence OSCA behavior and DBLR among Mainland Chinese consumers. Three notable findings were revealed, including (i) the positive effect between the wait for lower prices and hesitation at checkout; (ii) the sequential mediation effects of hesitation at checkout and OSCA; and (iii) perceived transaction inconvenience as a non-negligible boundary condition that strengthens the relationship between the wait for lower prices and hesitation at checkout.

Although this study provided several theoretical and practical implications, some limitations could not be avoided. First, we used a cross-sectional questionnaire to capture respondents' online shopping experience in Mainland China. Future research could incorporate an experimental design to better assess consumers' OSCA behavioral response. For example, by building realistic shopping environments where respondents operate the online shopping process in their natural state, scholars can assess inhibitors of OSCA that arise at any time by monitoring respondents' clickstream data or face-to-face communication (Rubin et al., 2020). Using experimental scenarios also allows the observation of differential responses by segmenting based on time (Li et al., 2021), product categories (Zhao et al., 2021), and selling platforms (Jiang et al., 2021), which would tap into more valuable information. In addition, future researcher can used experimental design to compared the different generations (Generation X, Generation Y, and Generation Z) toward the perception of OSCA and how it could have different behavioral outcomes on OSCA and intention to visit brick-and-mortar retail stores.

Second, retailers can use emerging technologies such as artificial intelligence to gain strategic momentum. For example, applying 3D gestures and service robotics to provide consumers with contactless smart services. This is because the actual use of contactless smart services like robots can positively affect the consumer experience (Guan et al., 2021). Therefore, when analyzing the OSCA phenomenon and intentions to visit brick-and-mortar retail stores, future research could analyze the importance of using technology as antecedents. The extension of capturing the complete consumers' behavioral decision through factors such as technology self-efficacy, warmth, and anthropomorphism will be interesting for future research.

Next, perceived transaction inconvenience was the only moderator used to explain hesitation to checkout. Therefore, we encourage future scholars to look into other potential moderating effects that may mitigate the relationship between the wait for lower prices and hesitation at checkout. For example, recent research by Cheah et al. (2020a) highlights that trust is an important factor to reduce the perceived complexity associated with online purchasing activities (BoŽič et al., 2020).

Finally, we also suggest for scholars to expand the current research model in other developing countries (e.g., Malaysia, Brazil) or in a cross-country scale to determine the purchasing behavior of online consumers in different cultural contexts.

## Data Availability Statement

The raw data supporting the conclusions of this article will be made available by the authors, without undue reservation.

## Author Contributions

SW, J-HC, and X-JL contributed to conception and design of the study. SW organized the database. J-HC performed the statistical analysis. SW wrote the first draft of the manuscript. YY, BN, X-JL, and J-HC wrote sections of the manuscript. All authors contributed to manuscript revision, read, and approved the submitted version.

## Funding

Shaoguan University cultivates researcher funding.

## Conflict of Interest

The authors declare that the research was conducted in the absence of any commercial or financial relationships that could be construed as a potential conflict of interest.

## Publisher's Note

All claims expressed in this article are solely those of the authors and do not necessarily represent those of their affiliated organizations, or those of the publisher, the editors and the reviewers. Any product that may be evaluated in this article, or claim that may be made by its manufacturer, is not guaranteed or endorsed by the publisher.

## Footnotes

^1^With an effect size of 0.15 and a 95% power level, a minimum sample size of 160 responses was required for the present study (Fink, 2017; Memon et al., 2020).

## Appendix

**Appendix 1:** Measurement items.

**No**.	**Items**	**References**
**Wait for lower price**		Kukar-Kinney and Close, 2010
1)	I decide to wait for the item to come on sale before buying it.	
2)	I decide that I may be able to find better sales at another online store.	
3)	I decide that I may be able to find better sales at a land-based store.	
**Hesitation at checkout**		Cho et al., 2006; Wong and Yeh, 2009
1)	I have hesitated to complete the checkout stage for selected items while shopping using my online device.	
2)	It has taken some time for me to click the final payment button to purchase products *via* an online device.	
3)	I have thought twice at the checkout stage for a purchase *via* an online device.	
4)	I have spent some time deciding whether to press the payment button in an online shopping task.	
5)	I have waited awhile thinking about whether to finish the checkout process for items in the final payment stage.	
**Online shopping cart abandonment**		Kukar-Kinney and Close, 2010; Huang et al., 2018
1)	How often do you place an item in the shopping cart, but do not buy it during the same session?	
2)	How often do you close the web-page, or log off the online shopping application before you buy the item(s) in your shopping cart?	
3)	How often do you abandon your online shopping cart?	
4)	How often do you leave items in your online shopping cart without buying them?	
**Decision to buy from a land-based retailer**		Rapp et al., 2015
1)	I search/view products online and then purchase products in the physical store.	
2)	I often visit from online shopping for products and examine product characteristics at physical store.	
3)	After visiting a product from online platform, I check the availability of products at the physical store and make a purchase at physical store.	
4)	I use a mobile internet device to fetch information about discount/promotion offers at physical stores.	
**Perceived transaction inconvenience**		Rajamma et al., 2009
1)	The online shop required me to register before making a purchase.	
2)	The order forms were very lengthy.	
3)	I got logged off in the middle and had to go through the entire process of completing information again.	
4)	Technical glitches in the site made the transaction difficult.	
